# A rare case of pseudomyogenic hemangioendothelioma (PHE)/epithelioid sarcoma-like hemangioendothelioma (ES-H) of the breast first misdiagnosed as metaplastic carcinoma by FNAB and review of the literature

**DOI:** 10.1186/s13000-019-0857-6

**Published:** 2019-07-17

**Authors:** Yan Ge, Xingtao Lin, Fen Zhang, Fangping Xu, Luqiao Luo, Weiye Huang, Zhihua Liu, Yanhui Liu, Zhi Li

**Affiliations:** Department of Pathology, Guangdong Provincial People’s Hospital/Guangdong Academy of Medical Sciences, 106, Zhongshan Road II, Guangzhou, 510000 China

**Keywords:** Breast, Pseudomyogenic hemangioendothelioma, Epithelioid sarcoma-like hemangioendothelioma, CD31

## Abstract

**Aims:**

Pseudomyogenic hemangioendothelioma (PHE)/epithelioid sarcoma-like hemangioendothelioma (ES-H) is a rare vascular tumor of intermediate malignancy that commonly occurs in soft tissue of distal extremities of young adults. PHE typically has a multifocal presentation and can involve several tissue planes, including the dermis, subcutis, muscle and bone.

**Methods and results:**

We present here a unique case of PHE/ESH that arose in the breast as well as a review of the published literature. The initial biopsy was interpreted as a metaplastic carcinoma. However, complete resection largely revealed plump epithelioid cells, and a more spindled cell component was also noted. The cells displayed abundant eosinophilic cytoplasm and central vesicular nuclei arranged in loose fascicles, with a mild, mixed acute and chronic inflammatory infiltrate. Overall, linear membranous staining of CD31 and lack of CD34 expression were highly suggestive of PHE. At the same time, FOSB immunoreactivity was observed, which supported PHE/ESH instead of metaplastic carcinoma. The patient has not shown recurrence in the half year follow up after total mastectomy.

**Conclusion:**

To our knowledge, this is the first report of breast involvement in this neoplasm. Recognition of its histopathological features and immunohistochemical reactivity will prevent misdiagnosis of breast lesions.

## Background

In 2013, Pseudomyogenic hemangioendothelioma (PHE)/epithelioid sarcoma-like hemangioendothelioma (ES-H) has been accepted as a new vascular fumor entity by WHO. PHE/ESH is a distinct, uncommon tumor with an endothelial phenotype that usually arises in soft tissue, and its biological behavior is intermediate between a benign hemangioma and a fully malignant angiosarcoma [1]. Histologically, PHE/ESH is characterized by ill-defined nodules of plump spindle and epithelioid cells with abundant densely eosinophilic cytoplasm that grow in sheets and fascicles [2]. PHE/ESH has no distinctive clinical features and is difficult to diagnose pathologically, especially when there is no architectural evidence suggestive of endothelial differentiation. At the same time, its vascular differentiation is essentially inapparent, with no well-formed vessels and only rare intracytoplasmic lumens, which makes this tumor type extremely difficult to diagnose. Approximately half of the patients in the largest published series were clinically misdiagnosed with other pathologies [3]. Therefore, increased awareness of this new entity is essential for both clinicians and pathologists.

In this article, we describe a rare case of PHE/ES-H of the breast first misdiagnosed as metaplastic carcinoma. To the best of our knowledge, this case represents the first case of PHE/ESH in breast. The present study demonstrates the diagnostic dilemma due to an exceedingly unusual location of PHE/ESH appearing as breast metaplastic carcinoma.

## Case presentation

In July 2018, a 43-year-old female patient presented to our clinic with the complaint of a mass and pain in her left breast. No significant signs were observed in her past medical and family histories. MRI revealed several masses on her left nipple, the lateral quadrant of the left breast, and the outer upper quadrant of the left breast. No palpable mass was detected in the other breast or axillae. Hence, a tru-cut biopsy was performed.

**Fig. 1:**
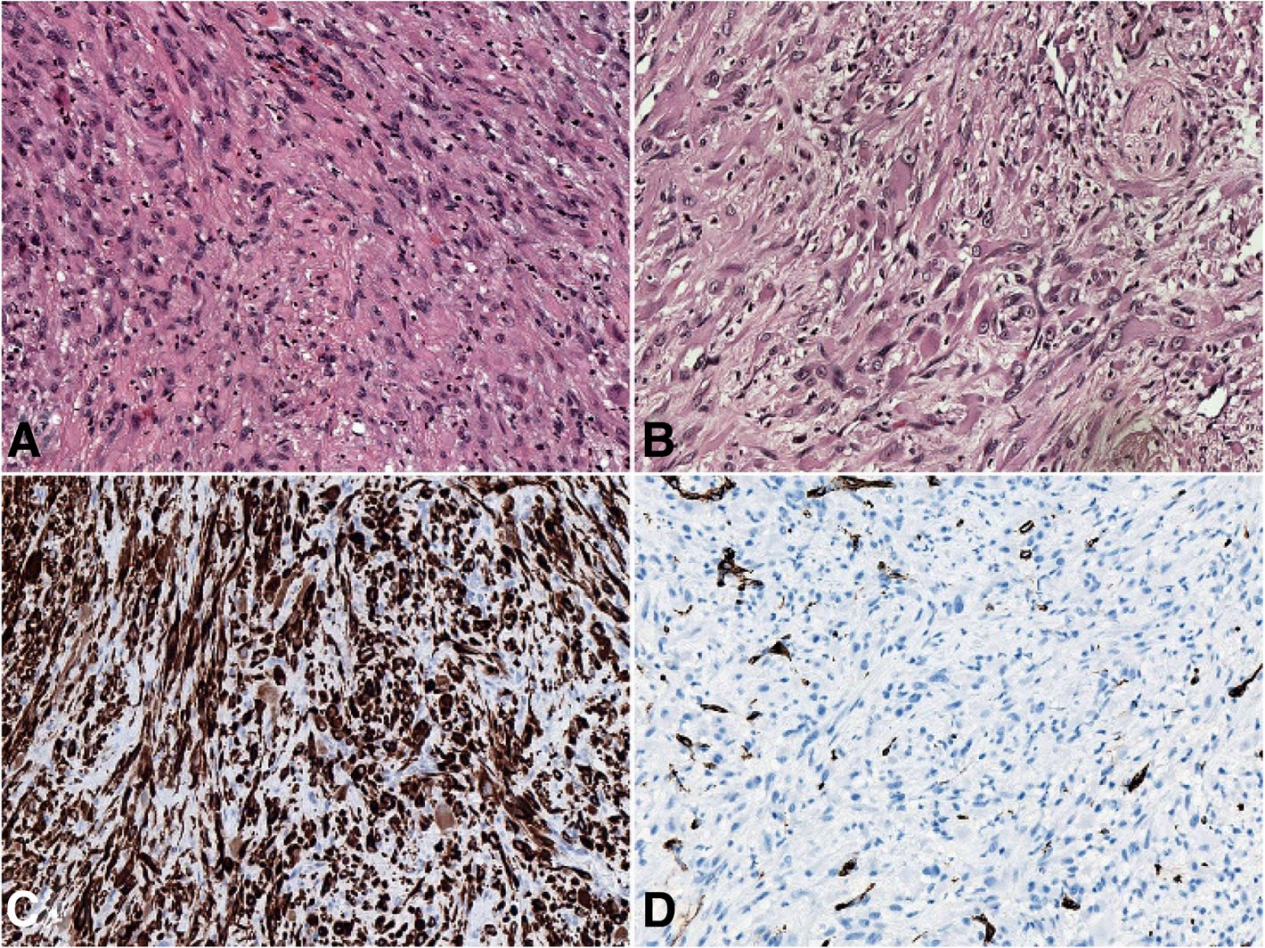
Microphotograph of tumor tissue by FANB. **a**. Loose fascicles of bland spindle cells with neutrophils scattered in the stroma (H&E, 200x). **b**. Rhabdomyoblast-like appearance of tumor cells with brightly eosinophilic cytoplasm (H&E, 200x). **c**. Strong extensive expression of AE1/AE3 in the neoplastic cells (200x). D.CD 34 were negative in tumor cells (200x)

The biopsy pathology revealed a solid, deep dermal and superficial subcutaneous mass consisting of relatively bland spindled cells with eosinophilic cytoplasm and moderately enlarged and hyperchromatic nuclei. Atypia and mitotic figures were inconspicuous. Acute and chronic inflammatory cells were present throughout the lesion; in particular, the stroma contained prominent neutrophil infiltration (Fig. 1a).

**Table 1:**
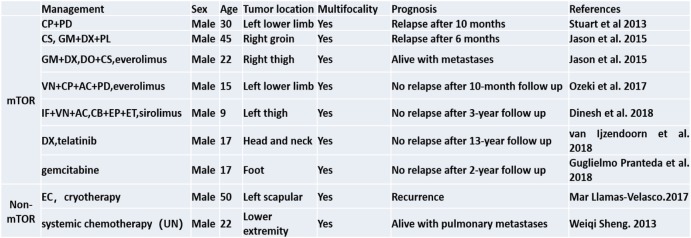
Different treatments for PHE/ESH in the literatures

Abbreviations: *CP* Cyclophosphamide, *PD* Prednisolone, *CS* Cisplatin, *GM* Gemcitabine, *DX* Docetaxel, *PL* Paclitaxel, *DO* Doxorubicin, *VN* Vincristine, *AC* Actinomycin, *IF* Ifosfamide, *EP* Epirubicin, *ET* Etoposide, *CB* Carboplatin, *EC* Electrocauterization, *UN* Unknown

**Fig. 2:**
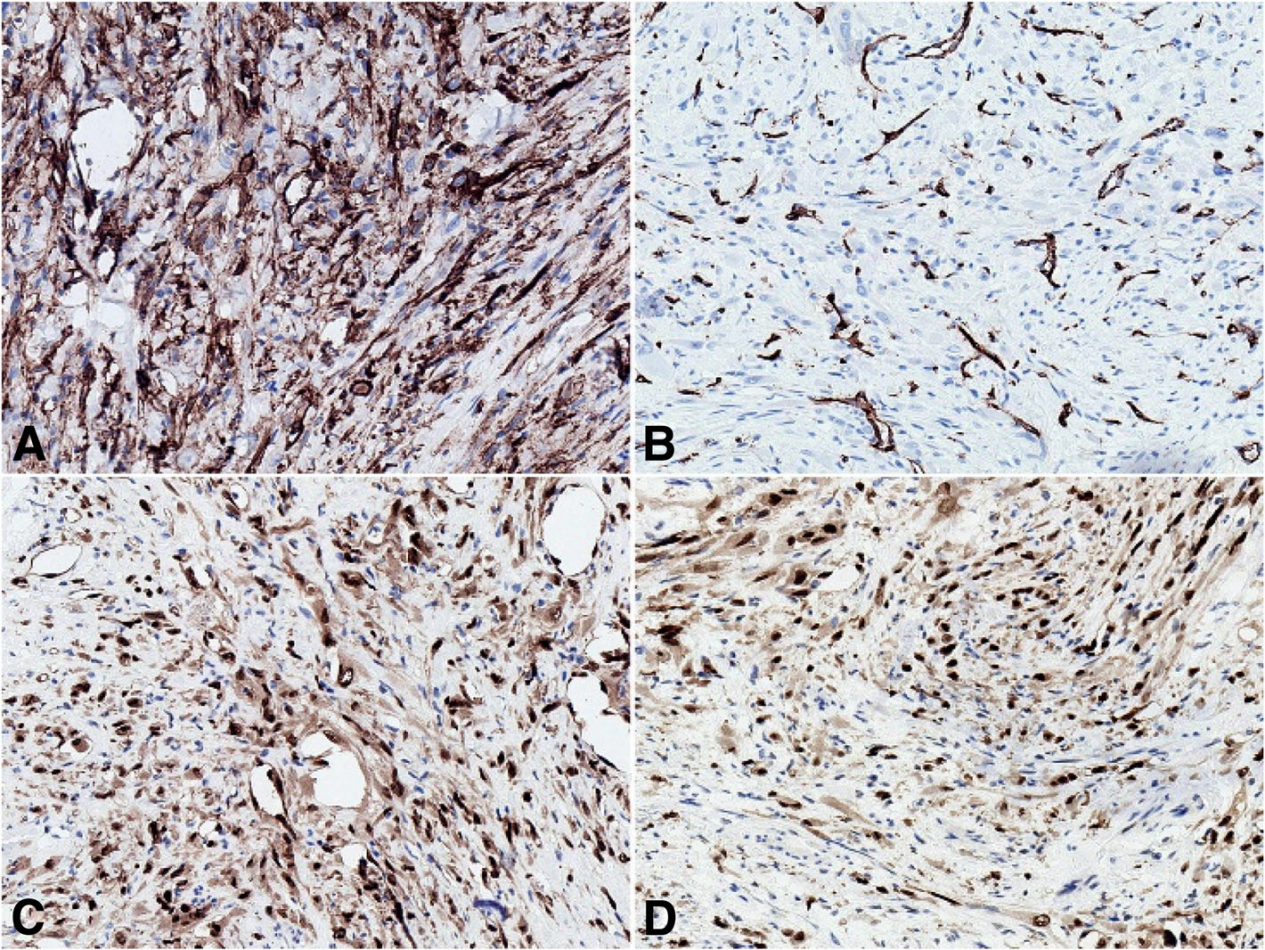
Immunohistochemical panel performed on the total mastectomy material. The neoplastic cells showed linear membranous staining of CD31 (**a**) and lacked of CD34 expression (**b**). The neoplastic cells showed strong and diffuse degrees of positivity for ERG (**c**)

**Fig. 3:**
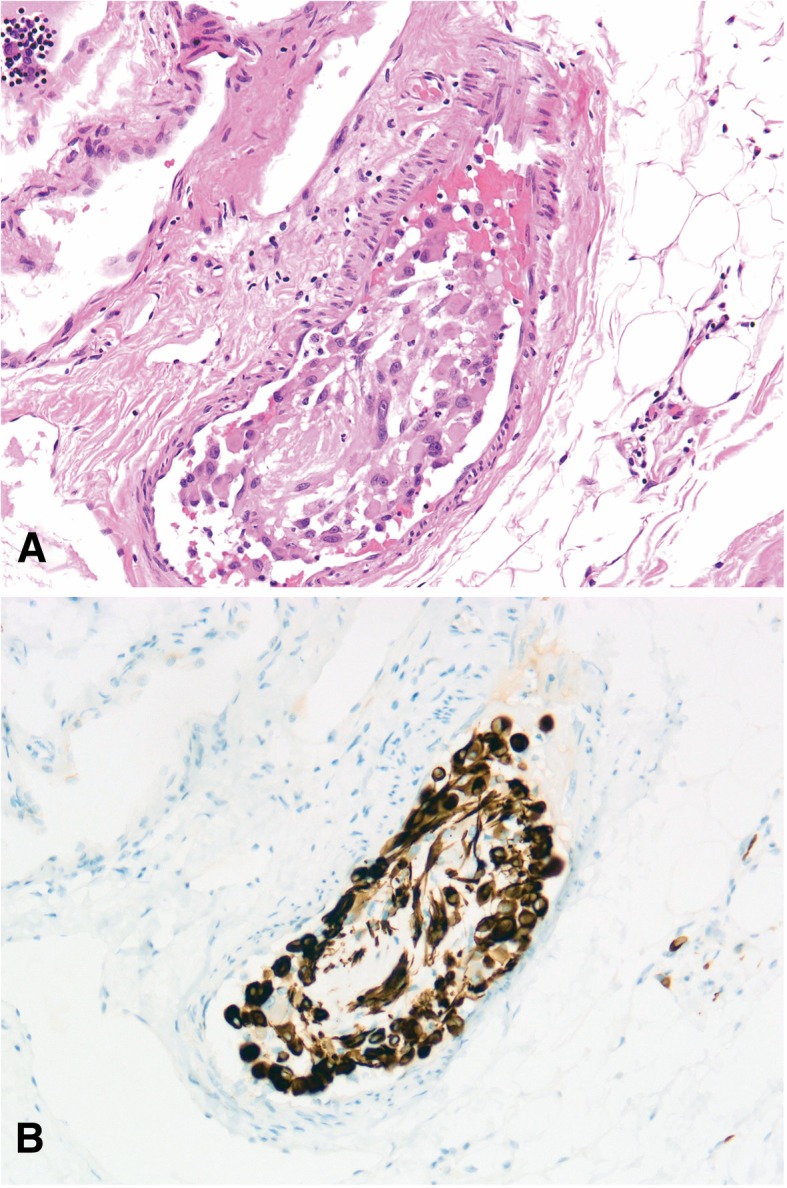
HE (**a**) and immunoreactivity of AE1/AE3 (**b**) showed a tumor cell embolus (200x)

The cells of interest were immunoreactive for AE1/AE3 (AE1/AE3, dilution 1:400; Gene Tech) and negative for SMA (1A4, dilution 1:1600; Gene Tech), Desmin (D33, dilution 1:200; Gene Tech), CD34 (QBEnd10, dilution 1:800; Gene Tech), ALK1 (ALK1, dilution 1:200; DAKO), S100 (2A10, dilution 1:400; IBL), β-catenin (E247, dilution 1:400; Gene Tech), and P63 (4A4, dilution 1:1000; Gene Tech). The Ki-67 (MIB-1, dilution 1:30; Biogenex) score demonstrated a low proliferation rate of tumor cells (1/10 HPF). The initial diagnosis was metaplastic carcinoma of the breast with no lymph node metastasis. Biomarker assessment revealed a triple-negative status. ER (SP1, dilution 1:1; Roche), PR (1E2, dilution 1:1; Roche) and c-erb-B2 (4b5, dilution 1:500; Ventana) were all negative. Total mastectomy was performed. The pathology was essentially the same as in the previous study, except for focal myxoid changes in the matrix. Moreover, the tumor cells exhibited a prominent epithelioid cytomorphology with a striking resemblance to rhabdomyoblasts (Fig. 1b). Therefore, more immunohistochemical analyses were performed, including for CD31 (JC70A, dilution 1:400; Gene Tech), FLi-1 (MRQ-1, dilution 1:100; ZATA), ERG (EPR3864, dilution 1:200; ZATA), INI-1 (25, dilution 1:200; ZATA), and FOSB (5G4, dilution 1:100; Cell Signaling Technology) (Fig. 2). AE1/AE3 analysis was repeated. The tumor cells were strongly and diffusely positive for AE1/AE3, FLi-1, ERG and FOSB. In situ hybridization for *TFE3* (Z-2109-50; ZytoVision) and *c-Myc* (05 J91–001; Abbott-Vysis) was also performed. Most of the cells were also positive for CD31, with a linear membranous pattern. The neoplastic cells maintained intact expression of INI-1. FISH results were negative for *c-Myc* amplification and for *TFE3* translocation*,* which ruled out epithelioid angiosarcoma (EAS) and epithelioid hemangioendotheliomas (EHEs). This immunophenotype supported the vascular nature of the neoplasm in tissue and the final diagnosis of PHE/ESH was made. Furthermore, tumor cell embolus and multiple lesions were found, and lactation surgery was not considered (Fig. 3).

## Discussion

In 1992, Mirra et al. [4] firstly described five cases of an unusual multifocal soft tissue tumor and called the unique lesions as “the Fibroma-like variant of epithelioid sarcoma”, describing it as a “fibrohistiocytic/myoid cell lesion often confused with benign and malignant spindle cell tumors”. In 2003, Billings et al. [3] described seven distinct cases of a low-grade vascular tumor and proposed renaming this tumor “epithelioid sarcoma-like hemangioendothelioma”, which was based on the presence of large cells with abundant eosinophilic cytoplasm upon microscopy with keratin positivity. Based on a series of 50 patients and the advent of newer immunohistochemical markers, Hornick and Fletcher [5–7] subsequently proposed changing the terminology to PHE/ESH, confirming its vascular origin and indolent behavior. In 2013, the current World Health Organization classification of soft tissue and bone listed PHE/ESH as an intermediate, rarely metastasizing, vascular tumor with peculiar clinical and pathological features [8].

In general, this neoplasm appears to be more common in males than in females (4.6:1) and typically occurs in men between 20 and 50 years of age. Clinically, PHE/ESH most commonly presents as multiple nodules in one anatomic region involving the soft tissues of the upper and lower extremities, but lesions may also arise in the trunk, spine, head, neck, bone and oral cavity [9–13]. Our patient is the first reported case of PHE presenting in the breast. There were several lesions on the nipple of the left breast, the lateral quadrant of the left breast, and the outer upper quadrant of the left breast. Histopathologically, PHE/ESH resembles a neoplasm with ill-defined nodules of plump spindle-shaped and epithelioid cells with abundant densely eosinophilic cytoplasm that grow in sheets and fascicles, sometimes mimicking rhabdomyoblasts. This infiltrative tumor often has a stromal neutrophilic infiltrate and sometimes also has a focal myxoid change in the matrix. Unlike other vascular tumors, PHE/ESH lacks multicellular vascular channels or intralesional hemorrhage. Cytologic atypia is typically mild to moderate, though rare cases have exhibited severe atypia. Mitotic activity is low, with most tumors having fewer than 5 mitoses per 50 HPFs or a mean mitotic rate of 2/10 HPFs [13]. Our case had a mean mitotic rate of 1/10 HPFs. The neoplastic cells usually express cytokeratin AE1/AE3, FLI-1, ERG and are negative for Desmin and S100. Furthermore, CD34 negativity is observed, which differentiates it from other vascular tumors such as epithelioid hemangioendothelioma or epithelioid angiosarcoma. Most notably, the tumor cells in our case were also positive for CD31, with linear membranous staining, which may facilitate diagnosis. Recently, the specific balanced translocation t (7; 19) (q22; q13) resulting in the fusion of the *SERPINE1* and *FOSB* genes was reported. This *SERPINE1- FOSB* gene fusion might lead to strong expression of *FOSB*, and identification of this genetic derangement is useful for diagnostic purposes [14, 15]. In our case, the tumor cells expressed CD31, AE1/AE3, FLi-1, and ERG and were negative for CD34. More importantly, FOSB overexpression was observed, which is consistent with the literature.

Oncologists are becoming more aware of PHE/ESH. However, it is still easily misdiagnosed. In fact, approximately half of the patients in the largest published series were clinically diagnosed with various other pathologies [6], such as epithelioid sarcoma, leiomyosarcoma, and EHE. These three tumors are particularly aggressive, and it is thus important to distinguish PHE. In general, ES, EHE and PHE share certain features: they all affect the young, show epithelioid and spindle cell morphology, and express FLI1 and ERG to varying degrees. PHE/ESH always has a neutrophil background, whereas the other two tumors are negative. Except for the immunophenotypic overlap, ES typically lacks reactivity for CD31 and lacks SMARCB1 (INI-1) expression, unlike PHE/ESH. The *WWTR1-CAMTA1* mutation is found in EHE, which is absent in PHE/ESH. Additionally, leiomyosarcoma shows reactivity to Desmin, Actin and Myogenin, but vascular markers are not expressed.

Considering all spindle cell lesions occurring in the soft tissues can occur in the breast with overlapping morphologies for different category of lesions so it is important to consider a wide differential diagnosis. In our case, it is easily to rule out diagnoses of inflammatory myofibroblastic tumor (IMT), aggressive fibromatosis, nodular fasciitis (NF), solitary fibrous tumor (SFT), since it is negative for ALK (5A4), β-catenin, SMA, Desmin, and CD34. But one of the most important differential diagnoses is metaplastic carcinoma. Our case was first misdiagnosed as metaplastic carcinoma because of its specific location and its immunochemical panel. Moreover, PHE/ESH has no unique radiological, the exact diagnosis can only be made on histopathologic examination. Neither fine-needle aspiration cytology nor core needle biopsy easily diagnoses PHE/ESH because it is difficult to obtain representative cells for a correct diagnosis by these techniques [16]. Histologically, both tumors share the same features, such as many spindle cells, mild to moderate nuclear atypia, and mitoses, with diffuse expression of keratins and lacking expression of ER, PR, and c-erb-B2. Metaplastic carcinoma, especially spindle cell carcinoma is characterized by atypical spindle cells, arranged in a multitude of architectural patterns raging from long fascicles in herringbone or interwoven patterns to short fascicles in a storiform pattern [17]. Infammatory infiltrate is often found in a proportion of cases, but usually with lymphocytes and dendritic cells not neutrophils. Clearly, vascular markers are not expressed in metaplastic carcinoma. Nonetheless, the diagnosis is not difficult if we are aware of this rare clinical entity in the breast.

PHE/ESH is a locally recurrent, rarely metastasizing tumor. A total of 82 patients with PHE/ESH have been reported, with follow-up available for 61 (74%); only 3 patients (5%) developed distant metastasis at 4, 8.5 and 16 years after the initial diagnosis. Almost half of patients had local recurrence or new lesions in the same region as the initial tumor, especially in the first year after diagnosis [18]. The efficacy of treatment is only partially known and still the object of study. Surgical excision is the first therapeutic choice for PHE/ESH, followed by chemotherapy or radiation. Most cases can be treated with wide local excision; however, amputation may be recommended for patients with extensive multifocal disease. Moreover, over one-third of patients exhibit relapse after surgery [19]. Based on this, systemic treatment is most likely necessary. Regardless, there are no guidelines because PHE/ESH is so rare. Different systemic therapies (Table 1) have been described in case reports in the literature. Among them, inhibitors of mammalian target of rapamycin (mTOR) show major efficiency [1, 11–13, 20, 21] in cases of progressive metastatic and relapsing multifocal PHE/ESH resistant to multiagent chemotherapy. mTOR, a serine/threonine kinase regulated by phosphoinositide-3-kinase (PI3K), acts as a master switch for numerous cellular processes, such as cellular catabolism and anabolism, motility, angiogenesis and growth. Several members of the PI3K/mTOR pathway have been implicated in the generation and propagation of vascular anomalies. As inhibitors of mTOR target protein synthesis downstream of the Akt pathway, they are predicted to be effective in disorders in which mTOR pathway-mediated growth control is affected. PHE/ESH is associated with the specific translocation t (7; 19) involving the *SERPINE1-FOSB* fusion gene. *SERPINE1* encodes a serine protease inhibitor family protein, known as plasminogen activator inhibitor-1 (PAI-1), which is reported to inhibit apoptosis by activating the Akt pathway [22]. Akt functions just upstream of mTOR and is overexpressed in endothelial cells of murine models of cutaneous vascular malformations. Therefore, inhibition of mTOR might constitute a target for therapy in the future. In our case, after total mastectomy, the patient did not show recurrence in the half year of follow-up.

## Conclusion

In summary, we present a unique case of PHE/ESH in the breast. Although extremely rare, PHE/ESH can present in the breast mimicking breast carcinoma. A high degree of suspicion is required to arrive at an accurate diagnosis. In view of the high incidence of local recurrences, continued close follow-up of the patient is mandatory.

## Data Availability

Data sharing is not applicable to this article as no datasets were generated or analyzed during the current study.

## Abbreviations

EAS: Epithelioid angiosarcoma
EHEs: Epithelioid hemangioendotheliomas
ER: Estrogen receptor
ES: Epithelioid sarcoma
ES-H: Epithelioid sarcoma-like hemangioendothelioma
Her-2: Human epidermal growth factor receptor-2
IMT: Inflammatory myofibroblastic tumor
mTOR: Inhibitors of mammalian target of rapamycin
NF: Nodular fasciitis
PAI-1: Plasminogen activator inhibitor-1
PHE: Pseudomyogenic hemangioendothelioma
PI3K: Phosphoinositide-3-kinase
PR: Progestogen receptor
SFT: solitary fibrous tumor
